# Fiber Link Health Detection and Self-Healing Algorithm for Two-Ring-Based RoF Transport Systems

**DOI:** 10.3390/s19194201

**Published:** 2019-09-27

**Authors:** Wen-Shing Tsai, Ching-Hung Chang, Zhin-Guei Lin, Dong-Yi Lu, Tsung-Ying Yang

**Affiliations:** 1 Department of Electrical Engineering, Ming Chi University of Technology, New Taipei City 24301, Taiwan; wst@mail.mcut.edu.tw; 2 Department of Electrical Engineering, National Chiayi University, Chiayi City 60004, Taiwan; tklingary@gmail.com (Z.-G.L.); xzericzx222666@gmail.com (D.-Y.L.);

**Keywords:** fiber Bragg grating, optical fiber transport system, optical add/drop multiplexer, self-healing

## Abstract

A two-ring-based radio over fiber (RoF) transport system with a two-step fiber link failure detection and self-healing algorithm is proposed to ensure quality of service (QoS) by automatically monitoring the health of each fiber link in the transport system and by resourcefully detecting, locating, and bypassing the blocked fiber links. With the assistance of the fiber Bragg grating remote sensing technique, preinstalled optical switches, and novel single-line bidirectional optical add/drop multiplexers, the optical routing pathways in the RoF transport system can be dynamically adjusted by the proposed algorithm when some fiber links are broken. Simulation results show that except in some extreme situations, the proposed algorithm can find the blocked fiber links in the RoF transport system and animatedly adjust the status of preinstalled optical switches to restore all blocked network connections, thereby ensuring QoS in the proposed RoF transport system.

## 1. Introduction

Radio over fiber (RoF) transport systems have been developed to support broadband wireless communication technologies, such as 5G and WiGig. Typically, RoF transport systems in ring topology can utilize wavelength division multiplexing (WDM) technology to extend the network capacity [1,2] and to provide a better self-healing function compared with tree-topology architectures to overcome fiber line failures [3,4,5,6]. However, if the ring-topology comprises a traditional optical add/drop multiplexer (OADM) [7,8], the transport system may be unable to exercise its self-healing function when a fiber link failure is present because both the added and passed optical signals in an OADM are transmitted in the same direction of the fiber ring [9]. To overcome such drawback, several single-line bidirectional OADMs (SBOADMs) [7,10,11] are developed based on optical multiplexer/de-multiplexer, multiport optical circulators (OCs), optical switches (SW), fiber Bragg gratings (FBGs), or optical amplifiers. Based on the published SBOADMs, the downstream light waves from central office (CO) can be transmitted to each SBOADM either in the clockwise (CW) or counterclockwise (CCW) direction of the fiber ring to prevent the blockage of fiber link. Nevertheless, when upstream light waves are added from the SBOADM to the ring-based network, they are transmitted along the same transmission direction of the downstream light waves; in this case, the upstream light waves are unable to avoid the impact of the blocked point. A backup fiber ring or SW is needed to reconfigure the optical passway in order to transmit the optical signals in either the general or backup fiber ring [12]. However, in normal conditions, the backup fiber ring is redundant. Besides, the complex and expensive devices inside the published SBOADMs can significantly increase the deployment cost and gradually introduce complications to the management of the network.

To address these bottlenecks, a novel SBOADM was proposed in our previous research [13] for building a large-scale optical fiber sensor network. Based on two FBGs, two three-port OCs, and three four-port OCs, the novel SBOADM can drop dedicated light waves from the fiber ring regardless of whether the light waves are transmitted in either the CW or CCW direction of the ring and can add and route upstream light waves back to the CO along the reverse transmission direction of the downstream light waves. No backup fiber ring is required in the network, and no dedicated optical multiplexer or optical switch is employed inside the SBOADM.

To further extend the application of SBOADM and to enhance its flexible routing ability and self-healing function for ring-based optical fiber transport systems, a two-ring-based RoF transport system with a self-healing functionality is built based on SBOADM. A dedicated algorithm, named two-step fiber link failure detection and self-healing algorithm, is also proposed to accurately determine the location of fiber line failure based on FBG remote sensing technique and to adjust the optical routing passway accordingly. FBG sensor can reflect specific wavelengths of light signals and the signals of the other wavelengths will penetrate directly. Its applications have been applied in various fields, such as geological hydrology, aerospace industry, pipeline monitoring, and so on [14,15,16,17,18,19]. Unlike other embedded self-healing architectures [6,20,21,22,23,24,25,26] that lack any algorithm or method for determining the breakpoint location, the proposed algorithm can locate two breakpoints and bypass the failure fiber links by adjusting the status of the pre-installed SWs in the proposed network.

## 2. Materials and Methods

**Figure 1:**
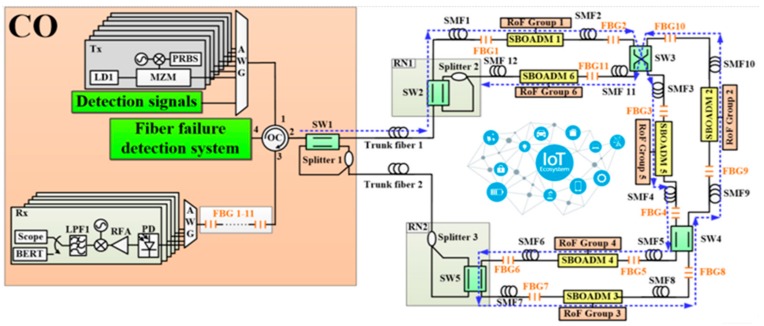
The proposed two-ring based radio over fiber (RoF) transport system with a self-healing functionality.

The proposed two-ring-based RoF transport system is shown in Figure 1, and its self-healing function was embedded by using SWs, FBGs, SBOADMs, and optical splitters. A total of 6 RoF groups located in 6 areas were connected to the CO by 2 fiber rings, 5 SWs, 3 optical splitters, 11 FBGs, and 6 SBOADMs. SW1, SW2, SW4, and SW5 were set to the parallel status, whereas SW3 was set to the cross status. The employed SWs and optical splitters allow the proposed transport system to establish additional optical pathways and to reconfigure the optical pathway in case of a fiber link failure. The 11 FBGs (FBG1–FBG11) deployed along with each span of single mode fibers (SMFs) were employed to remotely sense the health of each fiber link. The fiber failure detection system in CO usually sends out 11 dedicated optical detection signals to the transport system, and each FBG sensor was supported to reflect a dedicated optical detection signal back to the CO along the reverse downstream transmission routing pathway. When all detection signals were reflected back to the CO, they were routed by a four-port OC and reflected again by another set of 11 FBGs before they were received by the fiber failure detection system. Apart from the detection system, SBOADMs were also employed to drop and add dedicated optical signals among the fiber ring and connected RoF groups. To avoid interference between the detection system and RoF transmission groups, the reflection wavelengths of FBGs should be far from the optical carriers of RoF groups. In the network architecture, the CO was located 20 km away from remote nodes 1 (RN1) and RN2, and the distance between each pair of SBOADM was maintained at 1 km. In this case, downstream optical signals were sent from the CO to SBOADM1 via SW1, a 20-km trunk fiber 1, SW2 in RN1, and a 1-km SMF1. The optical signal belonging to RoF group 1 was dropped by SBOADM1. The other downstream optical signals passed through SBOADM1 and directed to SBOADM5, SBOADM4, SBOADM3, SBOADM2, and SBOADM6 sequentially.

**Figure 2:**
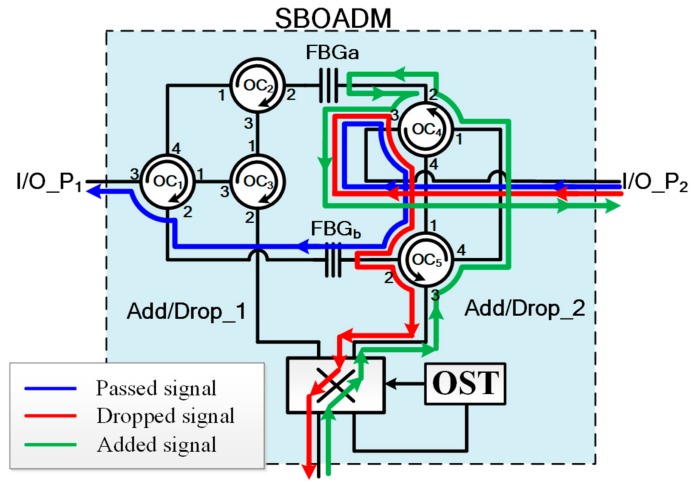
Structure of a single-line bidirectional optical add/drop multiplexer (SBOADM; the optical signals are fed from the right-hand side).

**Figure 3:**
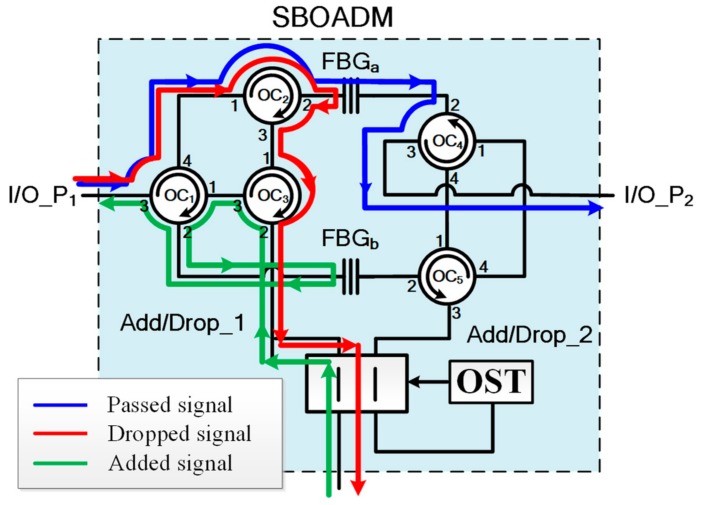
Structure of SBOADM (the optical signals are fed from the left-hand side).

To drop dedicated RoF signals into the connected RoF groups in both CW and CCW directions, the SBOADM published in [13] was modified as shown in Figure 2. A 2*2 SW and optical switch trigger (OST) were inserted into each SBOADM to bridge the two add/drop ports with the connected RoF group. The OST is a simple optical receiver model that can output high voltage when receiving optical signals and output low voltage when no optical signal is fed. For example, when the downstream light waves were fed into the SBOADM from the right-hand side, the optical pathway of the dropped, passed, and added optical signals are indicated by the red, blue, and green auxiliary lines, respectively, in Figure 2. In case the optical pathways were reconfigured due to fiber link failure, the downstream light waves were fed into the SBOADM from the left-hand side as shown in Figure 3. The dropped light waves were initially directed toward the OST because the SW in SBOADM is in the cross status. These light waves stimulate the OST to output a high-voltage pulse that changes the SW status to parallel status. Afterward, the downstream light waves can be dropped to relative RoF groups, and the added light wave can be sent back to the CO along the reverse transmission direction of the downstream light waves. When the optical pathways are reconfigured again, the light waves were fed into the SBOADM from the right-hand side. The dropped light wave was also fed into the OST along the parallel status SW, after which the status of SW changes back to cross status. Consequently, the dropped light wave can be redirected to the connected ROF group as shown in Figure 2.

## 3. Two-Step Fiber Link Failure Detection and Self-Healing Algorithm

### 3.1. First Detection Step of the Proposed Algorithm

To ensure quality of service (QoS), a two-step fiber link failure detection and self-healing algorithm was developed to monitor the fiber link health, locate the failure fiber links, and bypass the impact of fiber link failures on the proposed transport system. To evaluate the algorithm, the commercial software, VPI transmission maker, was employed to simulate the signal transmissions in the two-ring-based RoF transport system. In the simulation setup, the insertion losses of OC and SW are 0.8 dB and 0.7 dB, respectively. The reflection ratio and reflection bandwidth of the employed 11 FBG sensors (FBG1–FBG11) are 99% and 20 GHz, respectively. The reflection center wavelengths of these FBGS are 1550.017 nm, 1550.417 nm, 1550.817 nm, 1551.217 nm, 1551.617 nm, 1552.017 nm, 1552.417 nm, 1552.817 nm, 1553.217 nm, 1553.617 nm, and 1554.017 nm, respectively. In this case, when optical signals insert from the input/output port1 (I/O_P1) of the SBOADM to I/O_P2 or from I/O_P2 to I/O_P1, they will suffer roughly 2.4 dB insertion loss which is caused by passing through FBG one time and OC three times.

**Figure 4:**
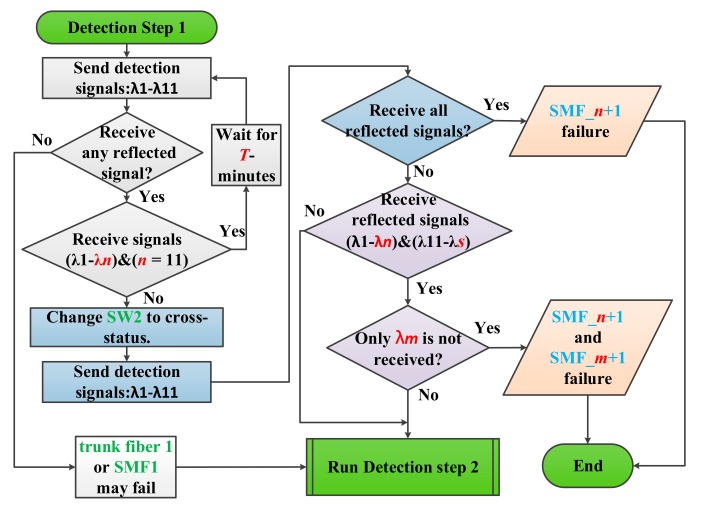
First detection step of the proposed two-step fiber link failure detection and self-healing algorithm.

**Figure 5:**
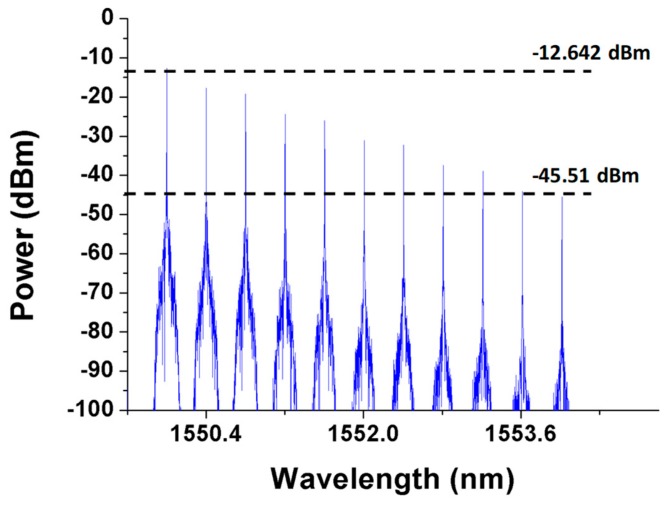
Detection signals received by the central office (CO) when no breakpoint is present in the transport system.

The first detection step of the proposed algorithm was designed to detect, locate, and bypass any fiber link failure except for trunk fiber 1. As shown in Figure 4, the CO periodically sends out 11 detection signals (λ1–λ11) every *T*-minutes to monitor the health of the two-ring-based RoF transport system in the detection phase (as indicated by the gray background in Figure 4). The preinstalled 11 FBG sensors (FBG1–FBG11) should be able to reflect each detection signal; otherwise, one or more fiber link failures may occur in the transport system. Generally, if detection signals send out the CO with 0 dBm, the reflected λ1 to λ11 in Figure 5 will suffer an attenuation of roughly 12.6 dB to 45.5 dB. Nevertheless, if none of the detection signals is received, then the trunk fiber 1 or SMF1 may reach failure. The detection system can execute the second detection step to find, locate, and bypass unknown fiber link failures as will be discussed later.

**Figure 6:**
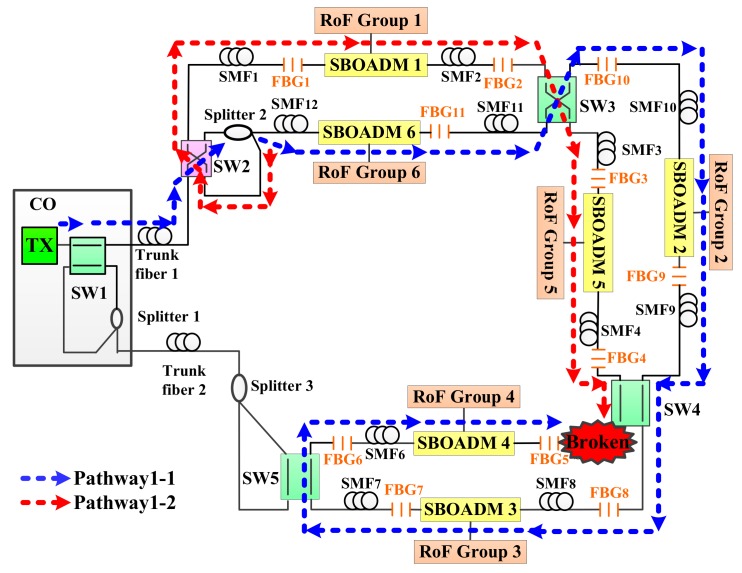
Routing pathways of downstream detection signals when the second phase of the first detection step is executed.

**Figure 7:**
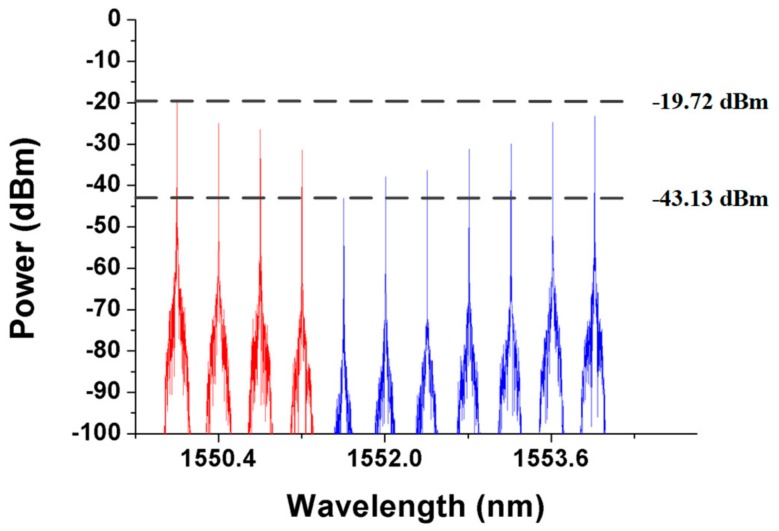
Detection signals received by the CO when the second phase of the first detection step is executed.

If parts of the detection signals are not received, that is, only λ1 to λ*n* are received and *n* < 11, then one or more fiber links may not function properly. The detection system can execute the second phase of the first detection step (indicated by the blue background in Figure 4) to locate and bypass any fiber link failure. In this step, the SW2 in RN1 switches to cross status and resends the 11 detection signals to the transport system. The CO can utilize low-power wide-area network (LPWAN) technique, such as long range (LoRa) or narrowband-internet of things (NB-IoT), to remote switch the SW2 status. The processes should be able to be finish in a few seconds since the response time of commercial SW is roughly 10 ms. For example, if the SMF5 does not work properly, as shown in Figure 6, only λ1 to λ4 will be received by the detection system in the first phase of the first detection step. In this case, *n* was set to 4. Subsequently, when SW2 switches from the parallel status to the cross status in the second phase of the first detection step, the downstream detection signals are split and transmitted by two pathways, namely, pathway1-1 and pathway1-2, in the RN1 as indicated by the blue and red auxiliary lines, respectively, in Figure 6. The optical signals in pathway1-2 would go through both SBOADM1 and SBOADM5, whereas those in pathway1-1 would go through SBOADM6, SBOADM2, SBOADM3, and SBOADM4 sequentially. All 11 optical detection signals would be reflected back to the CO as presented in Figure 7. In other words, the impact of the failure fiber link would be bypassed, and the breakpoint could be found at SMF*n*+1 (*n* = 4), which is consistent with our assumptions.

The first step of the proposed algorithm can deal with any fiber link failure except for that of trunk fiber 1. When trunk fiber 1 or any other two fiber links are blocked, switching only SW2 cannot heal the transport system and parts of the detection signals cannot be reflected back to the CO. To address these problems, the third phase of the first detection step (indicated by the purple background in Figure 4) was executed. In this phase, the detection system checks whether the two blocked fiber links are located on both sides of the SBOADM or not. The CO may only receive λ1 to λ*n* and λ11 to λ*s* (*s* ≥ 1). λ1 to λ*n* were reflected by pathway1-2, whereas λ11 to λ*s* were reflected by pathway1-1. If two failure fiber links are present in both sides of the SBOADM, then only λ*m* would not be received by the CO and the breakpoints could be found at SMF*n*+1 and SMF*m*+1. For instance, the SMF6 in Figure 6 is also broken simultaneously, and only λ1 to λ4 and λ11 to λ6 would be reflected back to the CO. Given that only λ5 was not received, the two failure fibers could be found at SMF*n*+1 (*n* = 4) and SMF*m*+1 (*m* = 5). Nevertheless, if more than one detection signal is not received by the CO, then the detection system should execute the second detection step of the proposed algorithm to determine the locations of breakpoints.

### 3.2. Second Detection Step of the Proposed Algorithm

**Figure 8:**
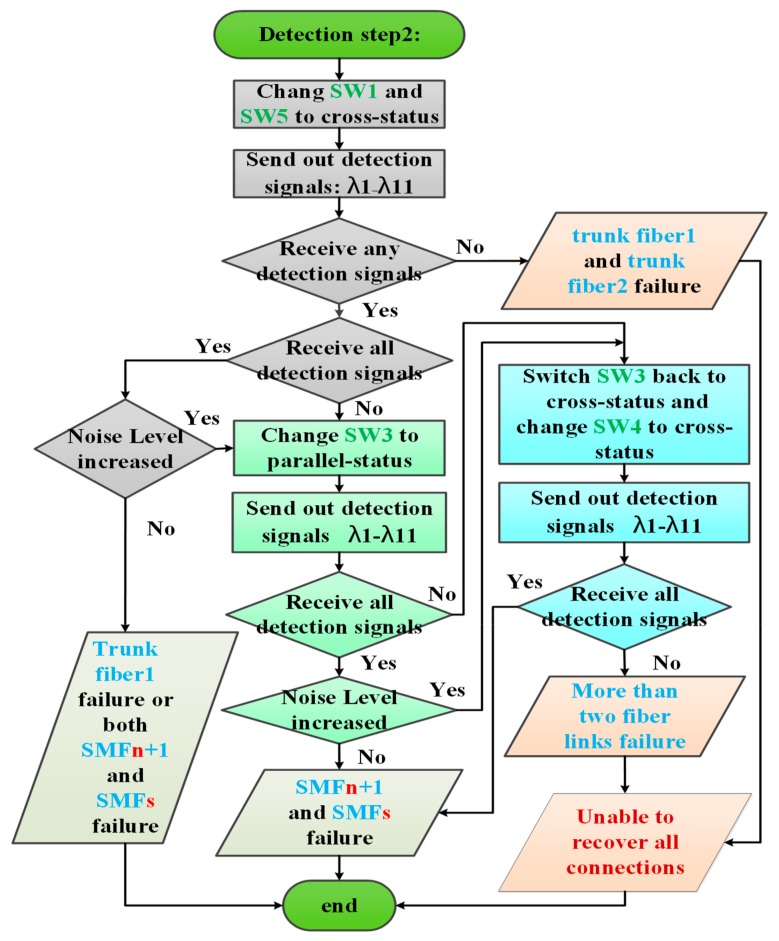
Second detection step of the proposed two-step fiber link failure detection and self-healing algorithm.

**Figure 9:**
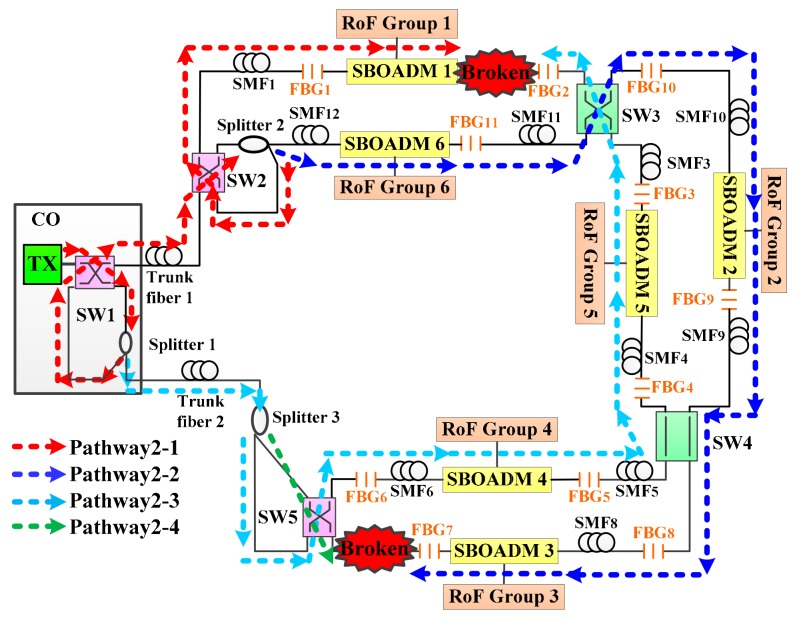
Routing pathways of the downstream detection signals during the execution of the first phase of the second step of the detection step.

**Figure 10:**
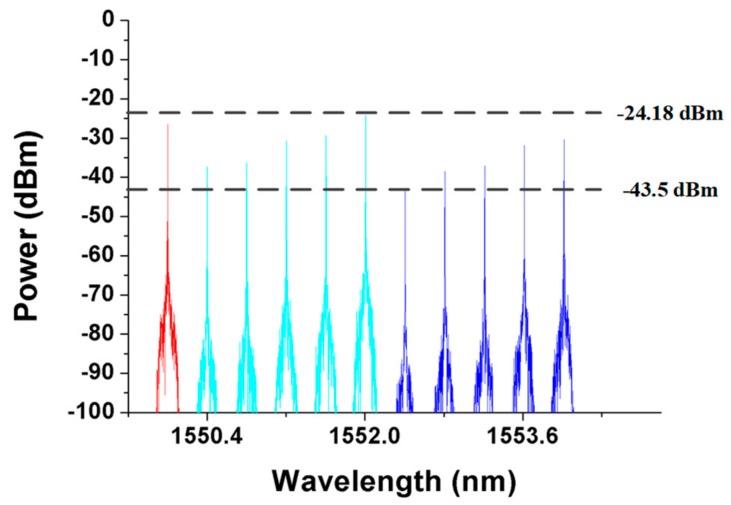
Detection signals received by the CO during the first phase of the second step of the detection step.

The flowchart of the second detection step of the proposed algorithm is shown in Figure 8. This step was mainly designed to deal with trunk fiber 1 failure and two fiber link failure conditions and would be executed after the first detection step. The first phase of the second detection step (indicated by the gray background in Figure 8) switched the SW1 in the CO and the SW5 in the RN2 from the parallel status to the cross status and then resent the 11 detection signals. In an extreme case, if both trunk fibers 1 and 2 are not functional, then no detection signal would be reflected by the FBG sensors. In this case, the two-step fiber link failure detection and self-healing algorithm could not recover all connections. Apart from the extreme case, if all detection signals were reflected back to the CO and the noise level of each received signal does not exceed that of the detection signals received in the first detection step, then the failure fiber link could be found at trunk fiber 1 or at both SMF*n*+1 and SMF*s*. Depending on the processing results obtained in the first detection step, if no detection signal was received, then the failure fiber could be found at trunk fiber 1. Otherwise, the failure fiber links could be found at SMF*n*+1 and SMF*s*, where *n* and *s* are defined in the first detection step. For instance, if SMF2 and SMF7 do not work, then *n* and *s* are set to 1 and 7, respectively, after executing the first detection step (only the SW2 changes to cross status). After executing the first phase of the second detection step, SW1 and SW5 switched to the cross status and the downstream detection signals were transmitted to pathway2-1, pathway2-2, pathway2-3, and pathway2-4 as shown in Figure 9. In this case, both the failure fiber links were bypassed, and the CO received all the detection signals as shown in Figure 10. The failure fiber links could be found at SMF*n*+1 (*n* = 1) and SMF*s* (*s* = 7).

In some cases, the CO still could not locate the failure fiber links even if all the detection signals were reflected back to the CO. Given that downstream detection signals go through the transport system along the four pathways when executing the first phase of the second detection step, parts of the downstream detection signals in some pathways may go through the two-ring-based RoF transport system and be injected into the CO without being blocked by any failure fiber link. In this case, the CO would receive all the detection signals, but the noise levels of these detection signals would be higher than those received in the first detection step. In this case, the second phase of the second detection step (indicated by the green and blue backgrounds in Figure 8) should be executed to locate the breakpoints. In parallel, if only parts of the detection signals were received by the CO in the first phase of the second detection step, then the second phase of the second detection step should also be executed to locate the breakpoints.

In the second phase of the second detection step, either SW3 or SW4 could be switched to determine the failure fiber link locations. For example, SW3 can be initially switched from the cross status to the parallel status before resending the detection signals to the transport system. If all detection signals were received, then the location of the failure fiber links could be found at SMF*n*+1 and SMF*s*; otherwise, SW3 could be switched back to the cross status and SW4 could be switched from the parallel status to the cross status. If all the detection signals were received by the CO, then the failure fiber links could also be found at SMF*n*+1 and SMF*s*. However, if the CO still could not receive all the detection signals, then more than two breakpoints might be present in the transport system and the two-steps fiber link failure detection and self-healing algorithm would be unable to recover all connections.

**Figure 11:**
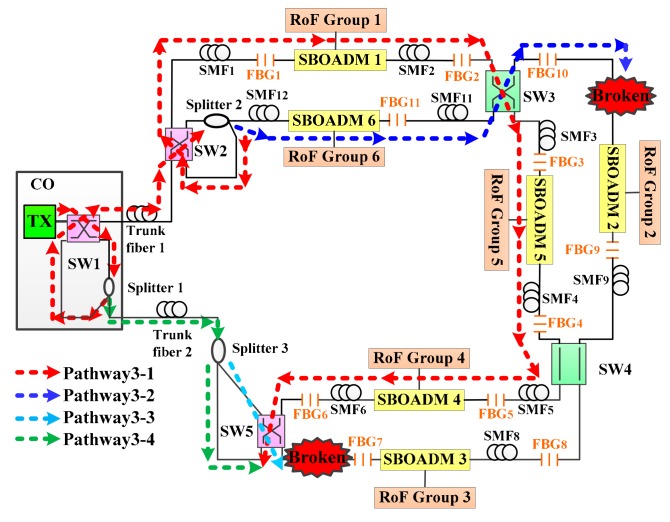
Routing pathways of the downstream detection signals when the first phase of the second detection step is executed.

**Figure 12:**
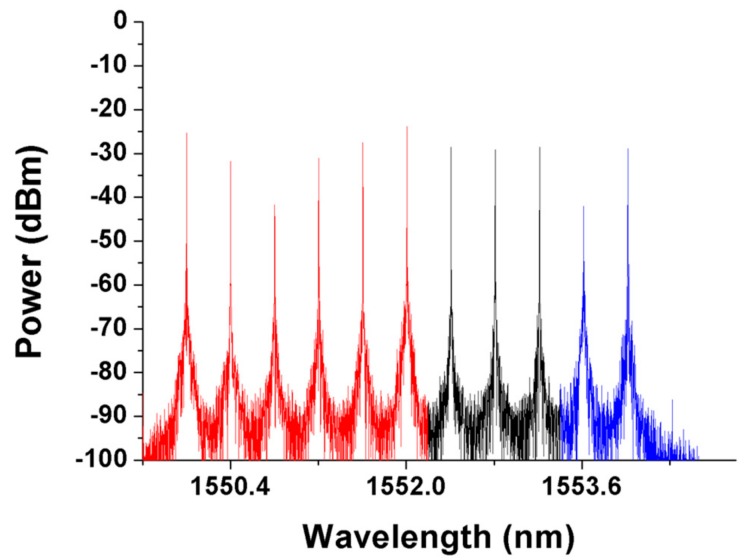
Detection signals received by the CO during the execution of the first phase of the second detection step.

**Figure 13:**
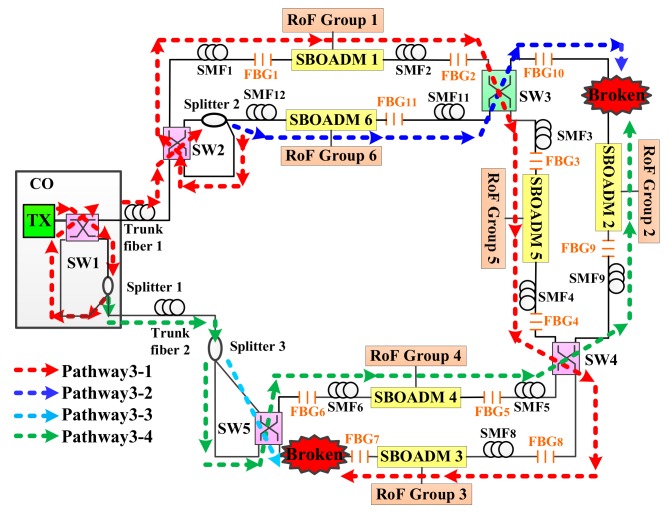
Routing pathways of downstream detection signals when the second phase of the second detection step is executed.

**Figure 14:**
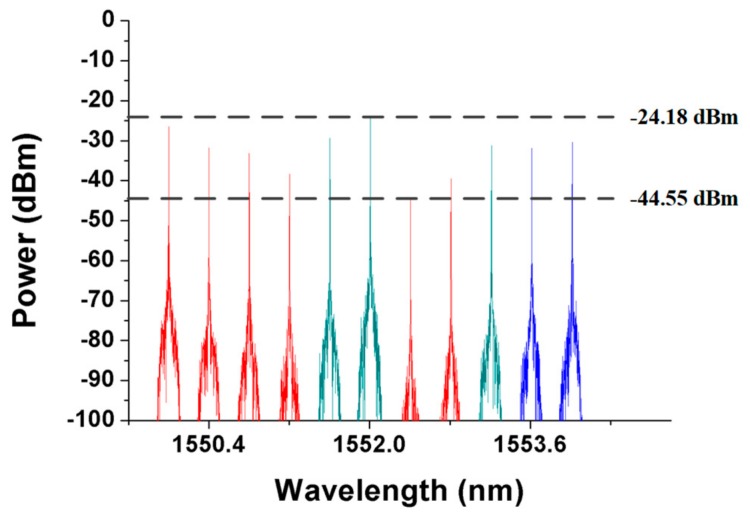
Detection signals received by the CO when the second phase of the second detection step is executed.

To evaluate the second phase of the second detection step, SMF10 and SMF7 were assumed to be blocked as shown in Figure 11. According to the first detection step of the proposed algorithm, the parameters *n* and *s* were set to 6 and 10, respectively. The CO received all the detection signals after executing the first phase of the second detection step. The optical spectra of the received detection signals are shown in Figure 12. The noise levels of the obtained detection signals were obviously larger than those shown in Figure 5 because the detection signals in pathway3-1 and pathway3-4 were transmitted through the RoF transport system and were directly routed back to the CO. The second phase of the second detection step should be executed to find the actual breakpoints. If SW3 in Figure 11 switched to the parallel status, then the detection signals in pathway3-1 and pathway3-2 would be routed back to the CO without being blocked by any failure fiber link. The noise level of the obtained detection signals would also be enlarged; therefore, SW3 should be switched back to the cross status and SW4 should be switched from the parallel status to the cross status as shown in Figure 13. The locations of all FBG sensors were reached by pathway3-1, pathway3-2, and pathway3-4. The optical spectra of the reflected detection signals in the CO are shown in Figure 14. Given that all the detection signals were received and their noise levels do not show any obvious increase, the blocked fiber links were assumed to be found at SMF*n*+1 (*n* = 6) and SMF*s* (*s* = 10).

The proposed two-ring-based RoF transport system with the assistance of the FBG remote sensing technique and the two-step fiber link failure detection and self-healing algorithm can automatically recover network connections in the presence of fiber link failures. Except in some extreme conditions, the proposed detection algorithm can locate up to two fiber link failures and restore all network connections before the blocked fiber links are repaired.

## 4. Conclusions

RoF transport systems have been utilized to develop a 5G mobile network. The network traffic loading and QoS requirements in a 5G system are much higher than those in a 4G environment. Providing RoF transport systems with self-healing functionality to prevent interruptions in network services is crucial. To contribute to the extant knowledge in this field, we develop a two-ring-based RoF transport system based on a self-developed SBOADM and a two-step fiber link failure detection and self-healing algorithm. With the assistance of the FBG remote sensing technique, a fiber failure detection system in CO can utilize optical detection signals to monitor the health of each SMF span in the RoF transport system. When one or more fiber link failures take place in the RoF transport system, the detection system follows the processes of the proposed two-step fiber link failure detection and self-healing algorithm to detect the failure fiber links and to bypass their impact. Simulation results show that apart from some extreme situations, the proposed algorithm can achieve its function properly when the blocked fiber links are less than three. Furthermore, the wavelength reflection ranges of the FBGs used in the SBOADMs are employed to drop or add RoF communication signals, but the FBG sensors were employed to detect the health of the fiber links only. The reflection center wavelengths of FBG sensors can be set apart from the range of communication wavelengths (optical carriers of RoF groups) to avoid interference with each other and the power levels of the detection signals can be adjusted to avoid non-linear effects such as four-wave mixing or stimulated Brillouin scattering (SBS). Although the grating pitch of the FBG sensors may vary with temperature or strain, increasing the FBG reflection window or adding a proper thermal control/protection will increase the stability of the proposed scheme. The proposed RoF transport system can then guarantee QoS by automatically detecting the location of blocked fiber links and recovering all network connections before repairing these links.
